# A cuproptosis-associated long non-coding RNA signature for the prognosis and immunotherapy of lung squamous cell carcinoma

**DOI:** 10.17305/bb.2022.8481

**Published:** 2023-08-01

**Authors:** Chunlan Hou, Xiuping Wu, Caoyang Li, Chao Wang, Jinbo Liu, Qing Luo

**Affiliations:** 1 Department of Clinical Medicine, Southwest Medical University, Luzhou, Sichuan, China; 2 Department of Cardiothoracic Surgery, The Affiliated Hospital of Southwest Medical University, Luzhou, Sichuan, China; 3 Department of Laboratory Medicine, The Affiliated Hospital of Southwest Medical University, Luzhou, Sichuan, China

**Keywords:** Lung squamous cell carcinoma (LUSC), cuproptosis, long non-coding RNA (lncRNA), prognosis, immunotherapy

## Abstract

Cuproptosis, a copper-induced mechanism of mitochondrial-related cell death, has been implicated as a breakthrough in the treatment of cancer and has become a new treatment strategy. Furthermore, long non-coding RNA (lncRNA) can change the biological activities of tumor cells. Globally, lung squamous cell carcinoma (LUSC) is one of the most difficult tumors to treat. As yet, nothing is known as to whether lncRNAs are related to cuproptosis in LUSC. Here, we developed a signature based on cuproptosis-associated lncRNAs that can predict the prognosis of LUSC and investigate the immunological features of LUSC. The Cancer Genome Atlas (TCGA) database was used to retrieve transcriptomic, clinical, and gene mutation data associated with LUSC. For statistical analysis, we utilized the R program. We created a signature consisting of three cuproptosis-related lncRNAs in this investigation (including AC002467.1, LINC01740, and LINC02345). Survival analyses and receiver operating characteristic (ROC) curves demonstrated that this signature exhibited powerful predictive capability. The predictive ability of the signature was confirmed by an ROC curve and principal component analysis; high-risk scores and high tumor mutation levels were associated with a reduced survival time. Tumor immune dysfunction and exclusion analysis further showed that individuals with low-risk scores may benefit from immunotherapy. The signature constructed by three cuproptosis-associated lncRNAs may represent a powerful prognostic marker for LUSC and may facilitate immunotherapy and provide a new direction for the treatment of LUSC.

## Introduction

Worldwide, lung cancer is one of the most difficult tumors to treat [1]. Approximately 85% of lung cancers are non-small cell lung cancers [2] and lung squamous cell carcinoma (LUSC) accounts for 20%–30% of this type of lung cancer [3]. Each year, LUSC kills approximately 400,000 patients globally [4]. The clinical prognosis of LUSC is poor, and no effective targeted therapy options are available [5, 6]. Therefore, there is an urgent need for a biomarker to predict the prognosis and personalized treatment of LUSC.

Researchers have identified many protein-coding mutations for targeted therapies by applying cancer exome sequencing and have found that the coding genome accounts for less than 2% of all sequences, thus suggesting that mutations within the non-coding genome can drive the development of cancer [7]. Long non-coding RNA (lncRNA) is a form of single-stranded RNA that is more than 200 bp in length [8]. Research has revealed that lncRNA plays a vital function in controlling gene expression as well as several other physiological and pathological processes [9–11]. Research has shown that lncRNA regulates transcription in cis or trans directions and the control of proteins or RNA in the body [12]. It has been reported that the function of lncRNA is similar to that of oncogenes, which control the occurrence and progression of a tumor through complex and precise regulation [13, 14]. There is evidence that lncRNA expression is abnormal in various tumors [15]. As a result, aberrant lncRNA expression can be employed as a biomarker of cancer progression [16–18]. In recent years, the role of lncRNAs has become increasingly investigated due to their role in the process of cancer [19]. In fact, lncRNAs have become emerging hotspots as diagnostic biomarkers or therapeutic targets for cancer therapy. A previous study explored the biological functions and pathways associated with lncRNAs and mRNAs by comparing their expression profiles in lung cancer; these researchers found that lncRNAs differed to a greater extent than mRNAs in lung cancer [20]. This could benefit the effective diagnosis and treatment of lung cancer.

Previous research has also demonstrated that an excess of copper can cause an accumulation of mitochondrial proteins, thus resulting in a distinct type of cell death [21]. Copper may cause cell death by a variety of methods, including apoptosis and autophagy, the formation of reactive oxygen species, proteasome inhibition, and anti-angiogenesis [22]. Abnormal copper homeostasis has been detected in many forms of malignant tumors [23, 24]. A number of cuproptosis-related genes have been shown to be associated with LUSC, such as NFE2L2, a prognostic marker for LUSC [25]. Furthermore, CDKN2A was shown to be closely associated with immune function in patients with LUSC; this gene was associated with an upregulated immune response in early-stage LUSC [26]. ATP7A, ATP7, SLC31A1, FDX1, DLAT, and LIA were recently identified as important biomarkers for treatment and prognosis in a multi-omics analysis of multiple cancers including LUSC [27]. However, it is still unknown whether cuproptosis-related lncRNAs affect LUSC. In this study, we created a signature consisting of three cuproptosis-related lncRNAs (including AC002467.1, LINC01740, and LINC02345). Survival analyses and receiver operating characteristic (ROC) curves demonstrated that this signature possessed powerful predictive capability. The predictive ability of the signature was confirmed by the ROC curve and principal component analysis (PCA); patients with a high-risk score and a high tumor mutation burden (TMB) level had a reduced survival time. Furthermore, tumor immune dysfunction and exclusion (TIDE) analysis showed that individuals with low-risk scores may benefit from immunotherapy.

This signature, based on three cuproptosis-associated lncRNAs, may represent a prognostic marker for LUSC, may contribute to immunotherapy, and provide a new direction for the treatment of LUSC.

## Materials and methods

### Data preparation and preprocessing

Our LUSC transcriptome analysis featured 49 normal samples and 502 tumor samples that were acquired from The Cancer Genome Atlas (TCGA) database (https://portal.gdc.cancer.gov/). We also obtained clinical and gene mutation information for LUSC from the TCGA database.

The inclusion criteria were as follows: (1) patients with squamous lung cancer, (2) patients with complete clinical information available in the TCGA database, (3) patients with lung squamous carcinoma of all ages, (4) patients with different clinical stages of squamous lung cancer, and (5) patients with squamous lung cancer of all genders and all races.

Patients were excluded if their clinical data was not complete.

Data was preprocessed with the Perl program (https://strawberryperl.com/, v5.30.0.1). We reviewed the literature related to cuproptosis [28–32] and identified 19 genes that can cause apoptosis through various copper-related pathways and defined them as cuproptosis-related genes: *NFE2L2, NLRP3, ATP7B, ATP7A, SLC31A1, FDX1, LIAS, LIPT1, LIPT2, DLD, DLAT, PDHA1, PDHB, MTF1, GLS, CDKN2A, DBT, GCSH, DLST*.

### Screening and evaluation of cuproptosis-related LncRNAs

Cuproptosis-related lncRNAs were defined as lncRNAs with sufficient correlation with 19 cuproptosis-related genes (| Pearson - |> 0.4 and *p* < 0.001). We performed co-expression analysis between lncRNAs in LUSC and 19 cuproptosis-related genes. According to statistical theory, a correlation coefficient of 0–0.09 refers to no correlation, 0.1–0.3 refers to weak correlation, 0.3–0.5 refers to moderate correlation, and 0.5–1.0 refers to strong correlation; we took a moderate correlation coefficient (0.4) as a screening criterion to obtain a suitable screening range. We employed “limma” software for co-expression analysis. The findings of the co-expression study were then generated by the “ggplot2,” “ggalluvial,” and “dply” software packages in R software.

### Establishment and confirmation of the cuproptosis-related LncRNA signature

First, LUSC tumor samples were divided into training and validation groups in a 1:1 ratio. Patients in the training group were used to construct the cuproptosis-related signature. All cuproptosis-related lncRNAs associated with prognosis were assessed by univariate Cox regression analysis (*p* < 0.05) and forest plots were generated. Prognosis-related lncRNAs were identified using LASSO regression analysis to narrow down the number of lncRNAs (using penalty parameters estimated by 10-fold cross-validation). We identified six cuproptosis-related lncRNAs that were strongly associated with the prognosis of LUSC patients. Finally, the three lncRNAs independently associated with prognosis were selected as the final models from the six lncRNAs by multifactorial regression analysis. In accordance with the median value of the risk score, the training group, the validation group, and all groups of patients were divided into high-risk and low-risk groups. The total risk score could be calculated using the following formula: risk score ═∑i=Expr(i)*LnCoef(i) in which Expr(i) represents the expression level of lncRNA, and LnCoef(i) represents the corresponding correlation coefficient. In both the training and validation groups, as well as all patients, survival analysis was used to compare the overall survival (OS) and progression-free survival (PFS) of the two groups. To verify the association between clinical characteristics and the signature, our team used the chi-square test. Independent variables were assessed by univariate Cox regression and multivariate Cox regression, respectively. ROC and Concordance-index (C-index) curves were used to assess the signature’s ability.

### Construction and calibration of the predictive nomogram

The “rms” tool in the R package was used to generate a hybrid nomogram that includes lncRNA signatures and clinicopathological factors. This nomogram was designed to estimate the OS (1-, 3-, and 5-years) of LUSC patients. Next, calibration curves were created to demonstrate the predictive capabilities of the established nomogram.

### PCA, gene ontology (GO) analysis, and Kyoto Encyclopedia of Genes and Genomes (KEGG) analysis

The R programs “limma” and “scatterplot3d” were used to depict the distribution of high-risk and low-risk group samples in PCA analysis. Gene functions were identified by GO enrichment analysis while KEGG analysis was used to identify potential biological signal pathways.

### TMB and tumor immune analysis

Next, we downloaded mutation data from the TCGA database; then, we used the “maftools” package in R software to check and integrate the TCGA data. We also investigated how TMB and survival varied across the two groups with different risk scores. We also used a waterfall map to express the association between the risk score of LUSC patients and somatic mutation. Finally, immune-related functions in LUSC were evaluated using a single-sample gene set enrichment analysis (GSEA) analysis and generated a corresponding heat map (*p < 0.05, **p < 0.01, ***p < 0.001).

### TIDE and immunotherapy

The TIDE database (http://tide.dfci.harvard.edu/login/) was used to generate a LUSC TIDE score file. In this study, we used the TIDE algorithm to predict the efficacy of immunotherapy. The greater the capacity of the immune system to escape, the less effective the immunotherapy.

### Expression levels of cuproptosis-related lncRNAs in LUSC patients

The lnCAR database (https://lncar.renlab.org/) contains 52,300 samples for differential expression analysis and 12,883 samples for survival analysis of ten cancer types [33]. We used experimental datasets from the lnCAR database to revalidate the level of the lncRNAs in the signature.

### Ethical statement

The data obtained from the public database are open access; thus, this study did not need the approval of a clinical ethics committee. The study complied with the corresponding rules of the public database.

### Statistical analysis

All statistical analyses in this study were performed using R project (https://www.r-project.org/, v4.1.3). Perl language (https://strawberryperl.com/, v5.30.0.1) was used for data processing. For the co-expression analysis, we used the “limma,” “ggplot2,” “ggalluvial,” and “dply” R packages. For model building, we used the “survival,” “caret,” “glmnet,” “survminer,” and “timeROC” R packages. C-index analysis was performed with “survival,” “rms,” “pec,” and “dplyr.” GO and KEGG analysis was performed with “clusterProfiler,” “org.Hs.eg.db,” “enrichplot,” “circlize,” “RColorBrewer,” “dplyr,” “ggpubr,” “ComplexHeatmap,” and “ggplot2.” We also used the “maftools” package to examine and integrate TCGA data and to perform correlation analysis between risk scores and somatic mutations in LUSC patients. We also used the “limma,” “GSVA,” “GSEABase,” “pheatmap,” “reshape2” packages to assess immune-related functions in LUSC. In addition, we used Wilcoxon’s test to analyze the differences in TMB between high-risk and low-risk groups. Prognostic values were tested using PCA, predictive column line plots, functional analysis, and Kaplan–Meier analysis. To compare categorical data between the groups, we used Chi-squared tests. *p* values < 0.05 were statistically significant (*p < 0.05, **p < 0.01, ***p < 0.001).

**Figure 1.**
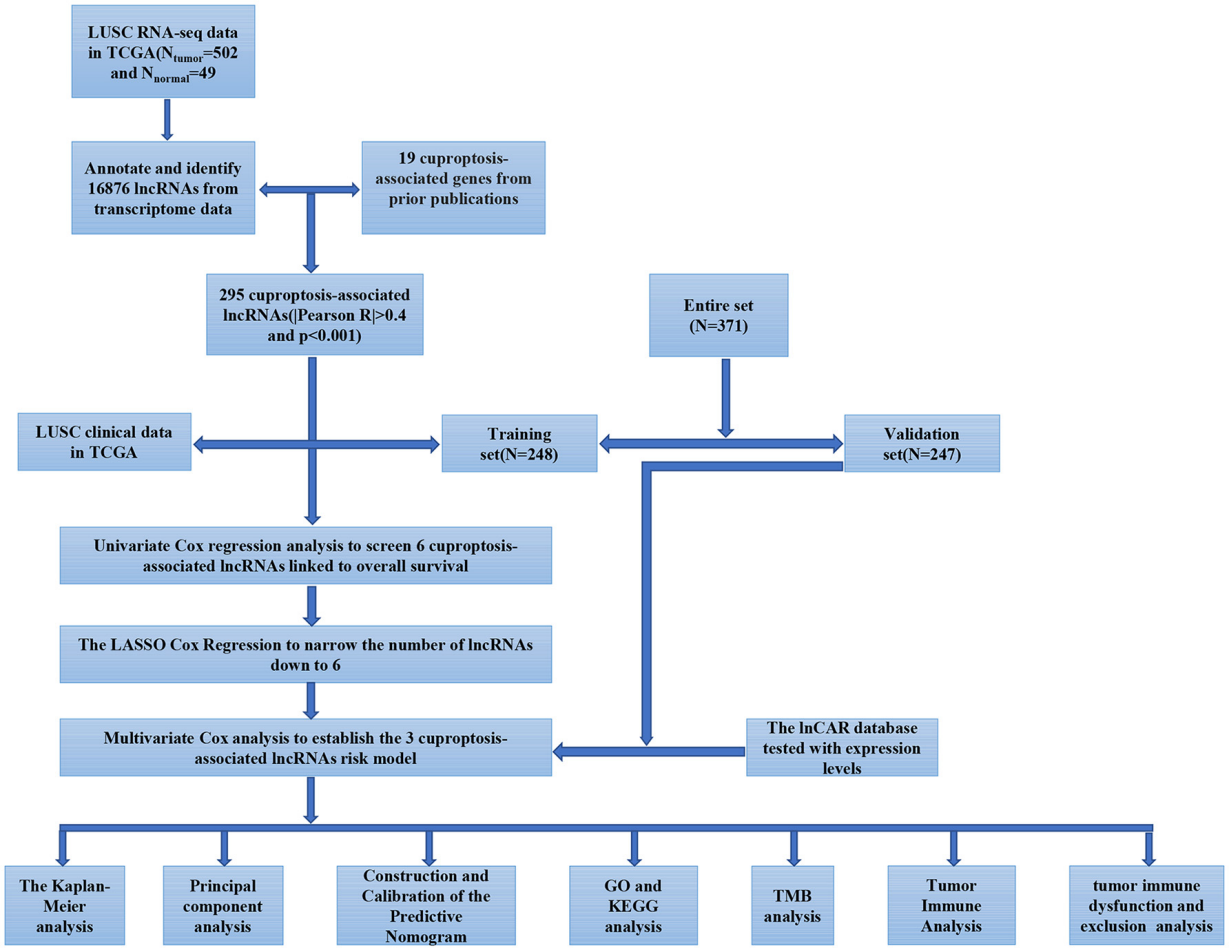
**Flowchart of the study.** The flowchart depicted the identification and validation of cuproptosis-associated lncRNA signatures. LUSC: Lung squamous cell carcinoma; OS: Overall survival. GO: Gene ontology; KEGG: Kyoto Encyclopedia of Genes and Genomes; TMB: Tumor mutational burden; lncRNA: Long non-coding RNA.

## Results

### Construction of the predictive signature

**Figure 2.**
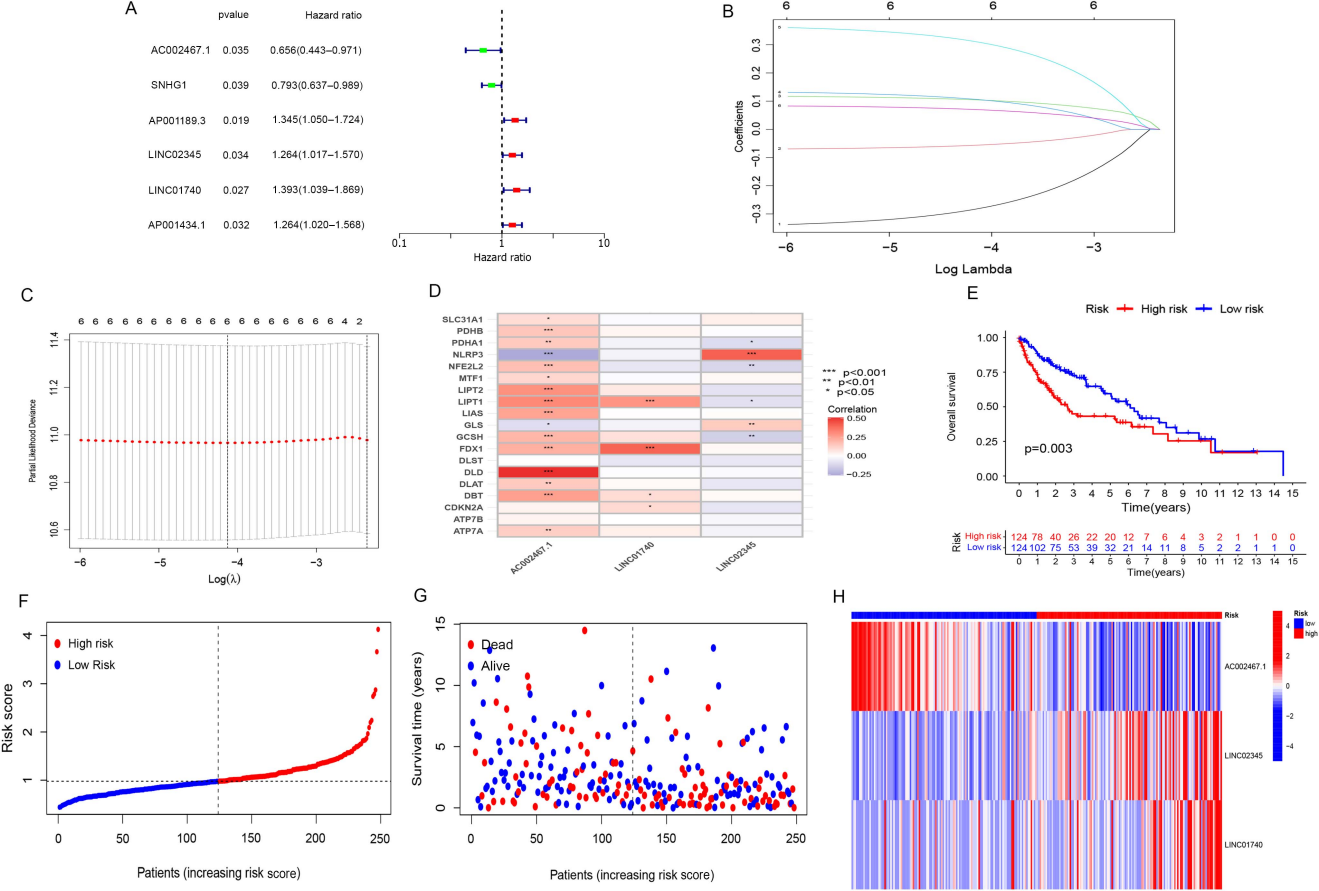
**Cuproptosis-related lncRNA prognostic markers in LUSC were identified.** (A) Prognosis-related genes of cuproptosis-related lncRNAs were shown in a forest plot; (B) The coefficients of cuproptosis-associated lncRNAs according to the determined tuning parameters (log λ); (C) LASSO identified 6 cuproptosis-associated lncRNAs based on the minimum criteria when the curve was at its lowest point; (D) In risk signatures, there is a correlation between lncRNAs and cuproptosis genes; (E) Survival analysis indicated that the OS of the patients with high-risk scores was worse (*P* ═ 0.003); (F) An analysis of the training group’s risk scores; (G) The training group’s survival status; (H) A heat map of three lncRNA expressions in the two groups. LUSC: Lung squamous cell carcinoma; OS: Overall survival; lncRNA: Long non-coding RNA.

Figure 1 shows a flowchart that describes the generation of the risk model and subsequent analysis. At the beginning of the study, we determined the homogeneity of the training and validation sets in terms of the baseline characteristics of clinical indicators and present the specific results in Table 1. In this study, 19 genes related to cuproptosis were collected by consulting literature. We used the “limma” package in R software for co-expression analysis to identify cuproptosis-associated lncRNAs (|correlation coefficient| > 0.4 and *p* < 0.001). In the training group, we identified six prognosis-related genes of cuproptosis-associated lncRNAs through univariate Cox regression analysis (Figure 2A). LASSO analysis is a common multiple regression analysis technique; we used a 10-fold cross-validation estimate of the penalty parameter to identify an optimal group of lncRNAs associated with prognosis (Figure 2B and 2C). Finally, three lncRNAs that were independently linked with prognosis were identified by multivariate regression analysis: AC002467.1, LINC01740, and LINC02345 (Figure 2D). The samples in the training group were divided into a high-risk group and a low-risk group in terms of the median risk score. According to survival analysis, patients in the high-risk group had a worse OS than the low-risk ones (*P* < 0.01; Figure 2E). Figure 2F illustrates the distribution of risk scores in the training group of patients with LUSC, and the survival status was shown in Figure 2G. In the risk heat map (Figure 2H), we observed that AC002467.1 was a low-risk lncRNA, and its expression decreased as the patient’s risk increased. In contrast, LINC02345 and LINC01740 were high-risk LncRNAs.

**Table 1:** Homogeneity of the training and validation sets in terms of the baseline characteristics of clinical indicators

**Covariates**	**Type**	**Total**	**Validation**	**Train**	***P* value**
**Age (years)**	<=65	189 (38.18%)	98 (39.68%)	91 (36.69%)	0.653
	>65	300 (60.61%)	148 (59.92%)	152 (61.29%)	
	Unknown	6 (1.21%)	1 (0.4%)	5 (2.02%)	
**Sex**	Female	129 (26.06%)	62 (25.1%)	67 (27.02%)	0.7018
	Male	366 (73.94%)	185 (74.9%)	181 (72.98%)	
**Stage**	Stage I	242 (48.89%)	120 (48.58%)	122 (49.19%)	0.7393
	Stage II	159 (32.12%)	84 (34.01%)	75 (30.24%)	
	Stage III	83 (16.77%)	38 (15.38%)	45 (18.15%)	
	Stage IV	7 (1.41%)	3 (1.21%)	4 (1.61%)	
	Unknown	4 (0.81%)	2 (0.81%)	2 (0.81%)	
**T**	T1	114 (23.03%)	56 (22.67%)	58 (23.39%)	0.4568
	T2	288 (58.18%)	142 (57.49%)	146 (58.87%)	
	T3	70 (14.14%)	40 (16.19%)	30 (12.1%)	
	T4	23 (4.65%)	9 (3.64%)	14 (5.65%)	
**M**	M0	407 (82.22%)	202 (81.78%)	205 (82.66%)	0.5352
	M1	5 (1.01%)	2 (0.81%)	3 (1.21%)	
	M1a	1 (0.2%)	1 (0.4%)	0 (0%)	
	M1b	1 (0.2%)	0 (0%)	1 (0.4%)	
	Unknown	81 (16.36%)	42 (17%)	39 (15.73%)	
**N**	N0	316 (63.84%)	166 (67.21%)	150 (60.48%)	0.4374
	N1	128 (25.86%)	62 (25.1%)	66 (26.61%)	
	N2	40 (8.08%)	16 (6.48%)	24 (9.68%)	
	N3	5 (1.01%)	2 (0.81%)	3 (1.21%)	
	Unknown	6 (1.21%)	1 (0.4%)	5 (2.02%)	

### Verification of the prognostic prediction model

**Figure 3.**
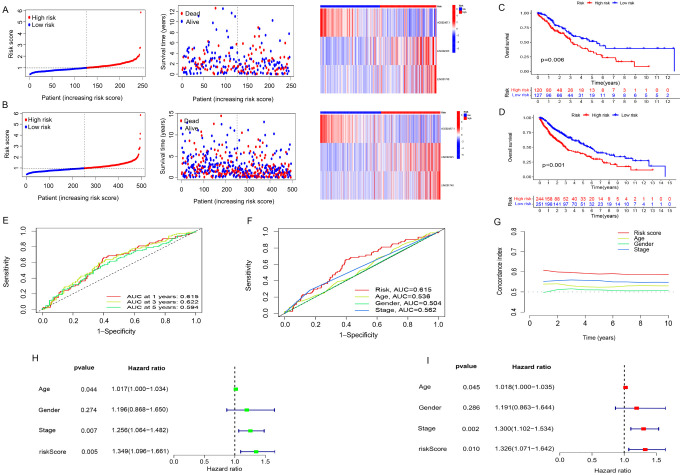
**Verification of prognostic prediction model.** (A) In the validation group, the risk score distribution, survival status, and risk heat map are shown; (B) Distribution of risk scores, survival status, and risk heat map across all cohorts; (C) In the validation group, the prognosis of the patient with a high-risk score was poorer (*P* ═ 0.006); (D) In all patients, the high-risk group had a worse prognosis (*P* < 0.001); (E) Accuracy of group-wide predicted risk signatures based on 1-, 3-, and 5-year curves; (F) Comparison of risk score prediction accuracy with clinicopathological parameters in age, gender, and stage; (G) C-index curve for risk signature. The risk score’s 10-year forecast accuracy was at its greatest; (H) Univariate regression analysis of prognostic factors; (I) Prognostic factor multivariate regression analysis. C-index: Concordance index.

We used the model built from patients in the training group to obtain the risk scores of the validation group and all patients. The patients in the validation group and all cohorts were then classified into two groups in line with the same median value. The risk score distribution, survival status, and risk heat map for the validation group are depicted in Figure 3A. The risk score distribution, survival status, and risk heat map for the entire cohort are given in Figure 3B. In the validation group (Figure 3C) and across all cohorts (Figure 3D), we detected the comparison of OS between the two groups. We found that the prognosis of the high-risk group was worse. Finally, we estimated the precision of cuproptosis-associated lncRNAs to predict a patient’s survival time using the area under the curve (AUC) value of the ROC curve. Patients with LUSC had AUC values of 0.615, 0.622, and 0.594 during the first year, the third year, and the fifth year, respectively (Figure 3E). The clinical-ROC curve (Figure 3F) and C-index data (Figure 3G) revealed that the model outperformed other clinical variables such as age, gender, and stage in predicting patient prognosis. In addition, used univariate regression analysis (Figure 3H) and multivariate regression analysis (Figure 3I) to show that the signature was an independent factor for predicting LUSC prognosis.

### Construction and assessment of the nomogram

**Figure 4.**
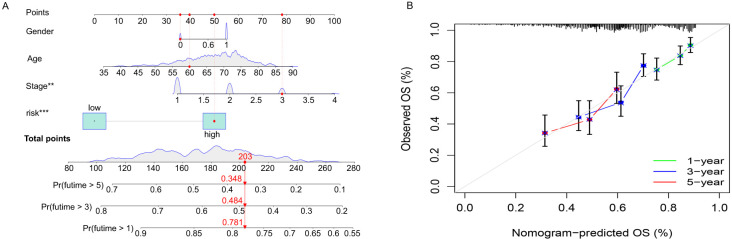
**Construction and assessment of the nomogram.** (A) The 1-, 3-, and 5-year survival rates; (B) Similarity between calibration and prediction results.

We combined the risk score with various clinicopathological features of LUSC to construct a hybrid nomogram model (Figure 4A). This nomogram can predict the 1-, 3-, and 5-year OS of patients with LUCS. A calibration plot demonstrated that the signature was similar to the ideal model, which ensured the signature performed well in predicting the prognosis (Figure 4B).

### PCA, GO, and KEGG analysis

**Figure 5.**
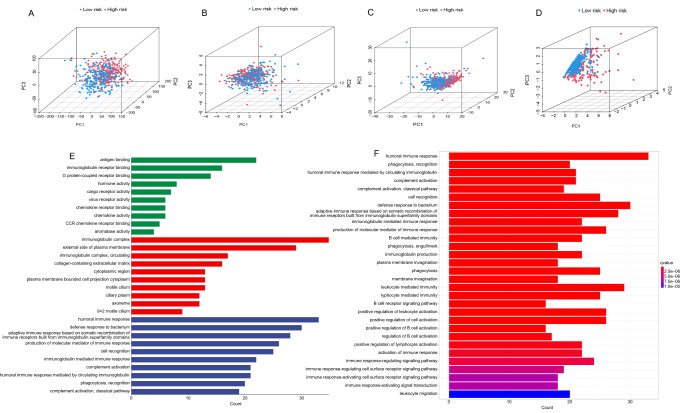
**PCA, GO, and KEGG analysis.** (A) The distribution of risk scores in terms of total gene expressions; (B) The distribution of risk scores in terms of cuproptosis genes; (C) The distribution of risk scores in terms of cuproptosis-associated lncRNAs; (D) The distribution of risk scores in terms of the signature; (E) Functional analysis; (F) KEGG pathway analysis uncovered significantly enriched pathways. PCA: Principal component analysis; GO: Gene ontology; KEGG: Kyoto Encyclopedia of Genes and Genomes.

The distribution of risk scores in terms of total gene expression profiles (Figure 5A), cuproptosis genes (Figure 5B), cuproptosis-associated lncRNAs (Figure 5C), and risk signature (Figure 5D) was then generated by PCA analyses. The signature performed best in discriminating and distinguishing the low-risk and high-risk groups.

According to GO analysis, functions related to the immune response process differed significantly between the high- and low-risk groups (Figure 5E). KEGG analysis showed that there were large differences in humoral immune response, bacterial defense response, and leukocyte-mediated immunity route when compared between the high- and low-risk groups (Figure 5F).

### TIDE and immunotherapy

**Figure 6.**
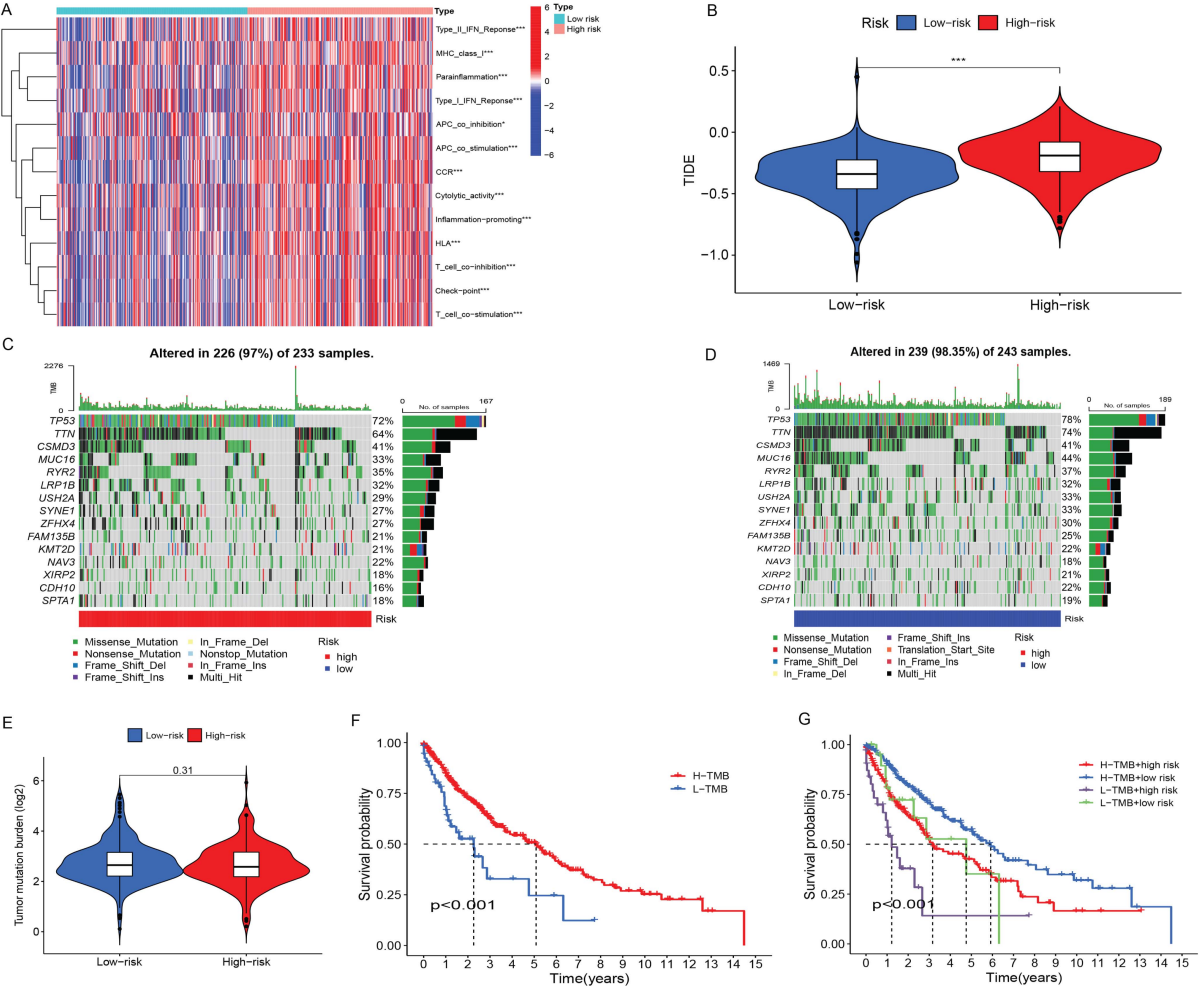
**Immunotherapy andTMB analysis.** (A) Immune function difference between two groups; (B) There was a substantial difference in TIDE scores between the two groups; (C) Mutated genes, mutation types, and mutation frequencies of the high-risk category; (D) Mutated genes, mutation types, and mutation frequencies of the low-risk group; (E) TMB expression levels between the two groups; (F) Survival analysis at different TMB levels; (G) The combined survival analysis of TMB and risk score. TMB: Tumor mutation burden; TIDE: Tumor immune dysfunction and exclusion.

Next, we explored the immune-related activities of the two groups of patients with LUSC (Figure 6A). Correlation analysis indicated that patients with different risk scores differed in terms of the following exemption functions: Type I IFN response, Type II IFN response, MHC class I, para inflammation, APC co-inhibition, APC co-stimulation, CCR, checkpoint, cytolytic activity, HLA, inflammation-promoting, T-cell co-inhibition, T-cell co-stimulation, and other functions.

**Figure 7.**
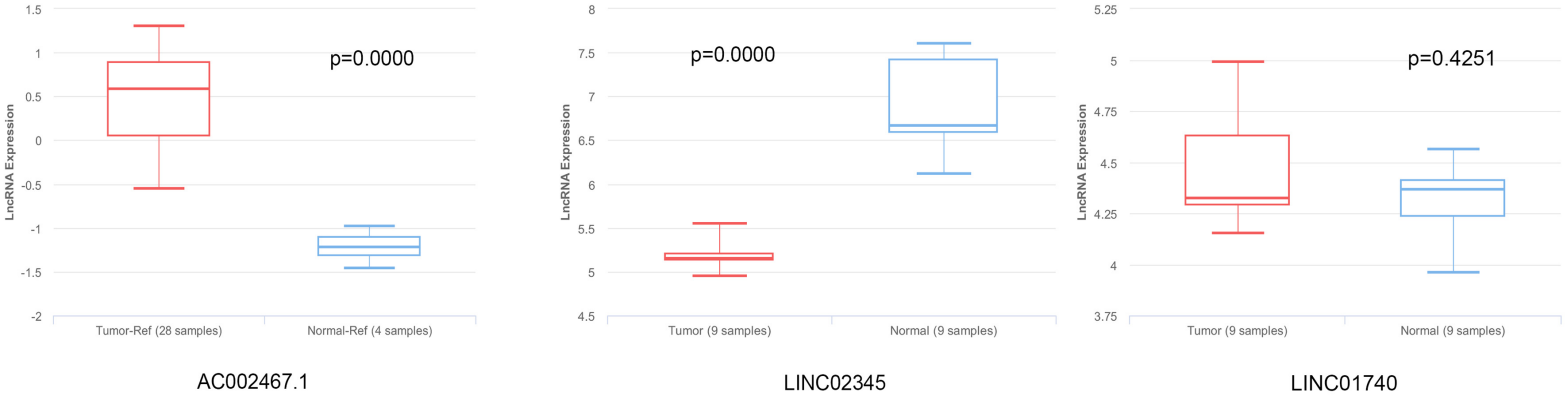
**Expression levels of cuproptosis-related lncRNAs in LUSC patients.** The levels of AC002467.1 and LINC01740 were higher in LUSC tissues compared with normal tissues, and the level of LINC02345 was lower in LUSC tissues. lncRNA: Long non-coding RNA; LUSC: Lung squamous cell carcinoma.

To compare the effectiveness of immunotherapy in the two groups, we used the TIDE algorithm. The TIDE score in the high-risk group was higher; this suggested that immunotherapy was more effective in low-risk patients (Figure 6B; *P* < 0.001).

### TMB analysis

We downloaded the mutation data associated with LUSC from the TGCA database and calculated the TMB levels for the two groups. Analysis revealed that TP53 was the gene with the highest mutation frequency in both the high-risk group (Figure 6C) and the low-risk group (Figure 6D). TMB expression levels were not statistically different when compared between the two groups (Figure 6E). The prognosis was better in patients with high TMB levels (Figure 6F). The combined survival analysis of TMB and risk score obtained a combined survival curve; this suggested that TMB and risk score had a significant effect on OS in patients with LUSC (Figure 6G).

### Expression levels of cuproptosis-related lncRNAs in LUSC patients

Figure 7 shows that the levels of AC002467.1 and LINC01740 were higher in LUSC tissues compared with normal tissues, and that the levels of LINC02345 were lower in LUSC tissues. These results were in line with those based on the TCGA datasets, suggesting that AC002467.1, LINC01740, and LINC02345 were key biomarkers of LUSC.

## Discussion

Our research is the first to investigate the involvement of cuproptosis-associated lncRNAs in the treatment of LUSC. To estimate the prognosis of LUSC patients, we created a cuproptosis-associated lncRNA signature that represents an independent risk predictor for LUSC prognosis. The PCA findings then revealed that the signature was the best at distinguishing between high- and low-risk patients. Furthermore, GO results revealed that the function of the immune response differed between the high- and low-risk groups, thus suggesting that LUSC may primarily affect the body’s immune system. KEGG analysis showed that humoral immune response and leukocyte-mediated immunity were the main biological pathways that differed between high- and low-risk groups. Numerous studies have shown that immunotherapy may be more successful for individuals with higher TMB levels because of the comparatively high number of neoantigens [34, 35]. TMB was greater in low-risk individuals according to our analysis of patient mutation data. Furthermore, patients with a high TMB level and a low-risk score possessed the longest survival time, according to a combined study of TMB and risk ratings. These findings show that immunotherapy may benefit low-risk individuals more. In addition, we discovered that the top three mutations in the high- and low-risk categories were TP53, TTN, and CSMD3. TIDE may be used for predicting immunotherapy responses by simulating the two primary pathways of tumor immune and escape [36, 37]. TIDE was used in the cohort to further illustrate the power of the signature in immunotherapy. Interestingly, we observed a positive association between TIDE and risk ratings, indicating that low-risk individuals may have better responses to immunotherapy. Finally, we validated our results using the lnCAR database.

LUSC is one of the most common subtypes of lung cancer [38]. Clinical investigations have indicated that the diagnosis and treatment impact of patients with LUSC is still not optimum at this time; this is the primary risk factor for the incidence and progression of lung cancer [39, 40]. As a result, it is vital that we identify a suitable biomarker to provide suggestions for the diagnosis, treatment, and prognosis of LUSC. Cuproptosis is a form of mitochondrial cell death triggered by copper [41]. Anticancer drugs are supposed to improve selectivity and reduce side effects; several copper ionophores have facilitated this area of research [42]. Furthermore, we discovered that lncRNA was linked to tumor invasion, metastasis, and prognosis [43]. Previous research has revealed that lncRNAs serve critical regulatory functions in the formation and progression of LUSC. For example, Zhang et al. discovered that lncRNA BBOX1-AS1 can promote LUSC proliferation and migration [44]. According to Peng et al. [45], the lncRNA PITPNA-AS1/miR-223-3p/PTN axis modulates the advancement of LUSC. Therefore, it is necessary to develop a cuproptosis-associated lncRNA model to predict the survival outcome and immune response of LUSC patients. The relevant lncRNAs identified in this study are gradually being reported in some cancers; for example, LINC01740 has been shown to be a potential prognostic biomarker in lung adenocarcinoma [46]. Also, LINC02345 is a prognostic marker in clear cell renal cell carcinoma [47]. Therefore, the lncRNAs in the signature may be able to identify future research directions for LUSC therapy. The novelty of our work is the combination of two factors (cuproptosis and lncRNA) to construct and validate a prediction model that provides an accurate and simple method for predicting patient survival and provides a theoretical basis for patient treatment.

However, our research has some limitations that need to be considered. First, these studies were carried out using data from a public database. Further experimental research needs to test and verify the created signature in order to increase the predictability of the outcomes. Second, we are unable to gather information on expression levels, OS, and follow-up data of additional lncRNAs that promote LUSC.

In summary, a predictive model based on three lncRNAs is a satisfactory indicator with which to assess the prognosis and immunotherapy of LUSC.

## Conclusion

The signature constructed by three cuproptosis-related lncRNAs can be used as prognostic markers of LUSC. This signature can play a role in immunotherapy and provides a new therapeutic direction for patients with LUSC.
